# MRI-Based Radiomics Analysis of Levator Ani Muscle for Predicting Urine Incontinence after Robot-Assisted Radical Prostatectomy

**DOI:** 10.3390/diagnostics13182913

**Published:** 2023-09-11

**Authors:** Mohammed Shahait, Ruben Usamentiaga, Yubing Tong, Alex Sandberg, David I. Lee, Jayaram K. Udupa, Drew A. Torigian

**Affiliations:** 1 Department of Surgery, Clemenceau Medical Center, Dubai P.O. Box 124412, United Arab Emirates; mshahait@yahoo.com; 2 Department of Computer Science and Engineering, University of Oviedo, 33204 Gijon, Spain; rusamentiaga@uniovi.es; 3 Medical Image Processing Group, Department of Radiology, University of Pennsylvania, Philadelphia, PA 19104, USA; yubing@pennmedicine.upenn.edu (Y.T.); jay@pennmedicine.upenn.edu (J.K.U.); 4 Temple Medical School, Temple University, Philadelphia, PA 19140, USA; alex.sandberg@temple.edu; 5 Department of Urology, University of California Irvine, Irvine, CA 92868, USA; davidleegum@yahoo.com

**Keywords:** radiomics, MRI, prostate cancer

## Abstract

Background: The exact role of the levator ani (LA) muscle in male continence remains unclear, and so this study aims to shed light on the topic by characterizing MRI-derived radiomic features of LA muscle and their association with postoperative incontinence in men undergoing prostatectomy. Method: In this retrospective study, 140 patients who underwent robot-assisted radical prostatectomy (RARP) for prostate cancer using preoperative MRI were identified. A biomarker discovery approach based on the optimal biomarker (OBM) method was used to extract features from MRI images, including morphological, intensity-based, and texture-based features of the LA muscle, along with clinical variables. Mathematical models were created using subsets of features and were evaluated based on their ability to predict continence outcomes. Results: Univariate analysis showed that the best discriminators between continent and incontinent patients were patients age and features related to LA muscle texture. The proposed feature selection approach found that the best classifier used six features: age, LA muscle texture properties, and the ratio between LA size descriptors. This configuration produced a classification accuracy of 0.84 with a sensitivity of 0.90, specificity of 0.75, and an area under the ROC curve of 0.89. Conclusion: This study found that certain patient factors, such as increased age and specific texture properties of the LA muscle, can increase the odds of incontinence after RARP. The results showed that the proposed approach was highly effective and could distinguish and predict continents from incontinent patients with high accuracy.

## 1. Introduction

Despite improvements in the perioperative outcomes of robot-assisted radical prostatectomy (RARP), postoperative urinary incontinence and erectile dysfunction still affect quality of life in a substantial number of prostate cancer patients. The urinary continence rate at 12 months post RARP varies from 68% to 90% [1]. The urinary continence rate after RARP is influenced by several factors, such as baseline continence, age, body mass index (BMI), prostate volume, surgeon experience, pre-existing lower urinary tract symptoms, and surgical technique [2].

The role of the levator ani (LA) muscle in the male continence mechanism remains unclear [3,4]. Several studies have shown an association between LA thickness and urinary continence rate after RARP [5,6]; conversely, other studies have found no such correlation [7].

Recently, interest in exploring the influence of skeletal muscle on other organs has grown. As a result, substantial evidence supports crosstalk between muscles and various organs, including the brain, adipose tissue, bone, liver, bowel, pancreas, vascular bed, and skin. Additionally, Mombiela et al. demonstrated that ultrasound radiomic features of skeletal muscles might correlate with organ dysfunctions such as myocardial infarction, dementia, and frailty. These findings suggest that the radiomic muscle phenotype may reflect cellular processes such as mitochondrial function. Most studies assessing the correlation between the LA muscle and continence post RARP have focused on its thickness in a single image. However, no studies have explored the association between LA muscle radiomic features and urine incontinence post RARP.

Therefore, we postulate that pre-operative magnetic resonance imaging (MRI)-derived radiomic features of the LA muscle might help identify patients with urine incontinence post RARP Identifying patients with such features associated with incontinence (and subsequently lower quality of life) could enhance preoperative counseling. In this study, we aim to characterize MRI-derived radiomic features of the LA muscle associated with urine incontinence in a cohort of men who underwent robot-assisted radical prostatectomy.

## 2. Materials and Methods

### 2.1. Study Population

This retrospective study was approved by the Institutional Review Board of our institution along with a Health Insurance Portability and Accountability Act waiver. We identified 140 patients who had undergone RARP for prostate cancer at a single institution between December 2013 and February 2018, where the procedure was performed by a robotic surgeon (DIL) using the same intra-operative technique, i.e., bladder neck sparing, maximal urethral sparing, suspension of dorsal vein complex, and bladder plication stitch. All patients underwent preoperative multiparametric MRI (mpMRI) and did not receive any neoadjuvant treatment or undergo any prior surgery. Patients were excluded if they did not have the outcome of interest or if the MRI images failed to pass the quality control steps (Supplementary Figure S1). Due to the exploratory nature of the analysis, the variability information needed to perform power calculation was not available; hence, power analysis could not be performed to estimate the sample size.

Patients were considered continent if they did not use any pads or liners. Early continence was defined as zero pads at 3 months plus one “safety pad”. Continence was defined as zero pads at 12 months plus one “safety pad”. In total, 62.9% of the patients were continent at 12 months and were labeled as the positive group (*n* = 61), while 37.1% were incontinent and were labeled as the negative group (*n* = 36).

### 2.2. Image Acquisition and Preprocessing

The MRI scans were previously acquired for clinical purposes on 1.5 and 3.0 Tesla clinical MRI scanners (Espree model, Siemens Healthcare, Erlangen, Germany). Coronal T2-weighted turbo spin-echo images through the pelvis varied from 256 × 256 to 512 × 512 in matrix size, with 18–40 slices acquired from the aortic bifurcation to the femoral heads, with an average slice thickness of 3 mm and 3.9 mm spacing between slices. The voxel size varied from 0.2 × 0.2 × 3.5 mm^3^ to 0.7 × 0.7 × 4.8 mm^3^. Supplementary Table S1 summarizes patient characteristics. As described previously, the images were trimmed to include the region of interest, corrected for inhomogeneity, and standardized such that the same tissue regions had similar image intensity meanings [8]. Subsequently, in each slice of the trimmed and standardized image data set, the LA muscle was manually identified and delineated using the CAVASS (Computer-Assisted Visualization and Analysis Software System) software system developed by the University of Pennsylvania’s Medical Image Processing Group [9]. Integrated with open-source toolkits such as ITK and VTK, CAVASS specializes in visualizing, processing, and analyzing 3D and higher-dimensional medical imagery.

After an extensive review of the literature, we decided to include the LA muscle components of the urogenital hiatus. Therefore, on coronal images, portions of the LA muscle surrounding the prostate gland from the posterior edge of the symphysis pubis to the posterior aspect of the prostate gland were segmented, including obliquely oriented parts superior to the anus and excluding the vertically oriented external anal sphincter, consistently in all patient images.

**Figure 1:**
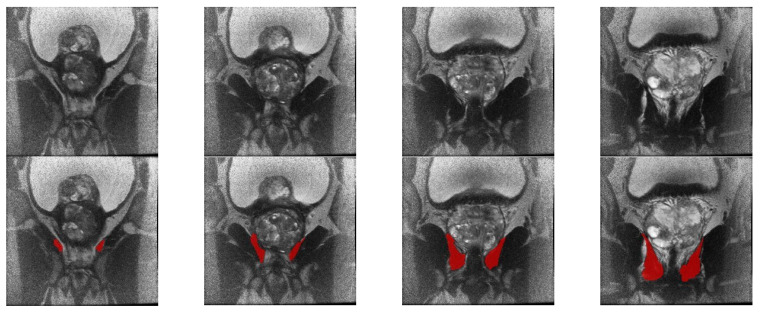
Visual appearance of the levator ani muscle region in the standardized coronal T2-weighted MRI image (**top** row) and the delineated mask (**bottom** row) for the region of interest.

**Figure 2:**
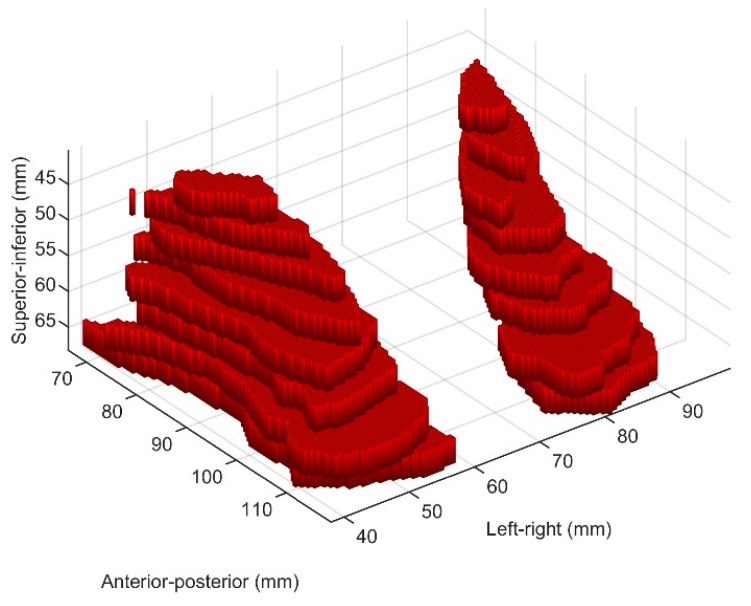
3D representation of the contoured levator ani muscle for one patient.

Figure 1 displays exemplary slices from one patient, where both the standardized MRI slice (top row) and the delineated LA region (bottom row) are shown. Figure 2 shows a 3D representation of the delineated LA region, where two large components of the LA region can be seen. This represents a 3D volumetric binary image.

### 2.3. Biomarker Discovery Approach

The biomarker discovery approach used in this study is based on the optimal biomarker (OBM) method [10]. Briefly, the proposed procedure first extracts a large set of quantitative features from the image region of interest (in our case, the LA region), including shape, size, intensity, and texture properties. Subsets of features were then selected based on the statistical importance of the considered outcome, and mathematical models were created using each subset of features. A particular subset of features was evaluated based on the ability of the model to predict the outcome, and the best subset was selected.

### 2.4. Feature Extraction

The proposed approach begins with extracting a set of quantitative features from the region of interest in the 3D MRI volume. Features were classified into four categories: morphological, intensity, texture, and clinical variables, such as age, prostate size, BMI, and urethral length. Morphological features describe the shape, size, and spatial organization of the region of interest in the volume. Global features include the volume and surface area. Subsequently, the features for each of the two components of the LA muscle, left and right, were calculated. These regions correspond to the two largest connected components in the 3D volumetric binary image. For each component, the volume, surface area, and eigenvalues obtained by Principal Component Analysis of the 3D object volume were calculated. The eigenvalues were proportional to the squares of the lengths of the equivalent ellipsoid axes for each component. Thus, they are generally used as approximations of the shape of a volume [11]. Supplementary Figure S2 shows a 3D representation of the equivalent ellipsoids. The linear size estimate is described by the variable slambda, defined as the square root of the sum of eigenvalues. Additional morphological features, including different ratios between the eigenvalues and the linear size estimate and between the left and right components, were calculated. Overall, thirty-five morphological features were calculated for each patient.

Intensity features are statistical measures that describe the intensity or brightness values of the voxels in the LA muscle. These features included mean, median, standard deviation, mode, maximum, minimum, quartiles, moments, skewness, kurtosis, and peak height. Overall, fourteen intensity features were extracted for each patient.

The texture features were designed to quantify the perceived texture (heterogeneity) of the LA muscle. Two different types of texture descriptors were used: the local binary pattern (LBP); and the gray-level co-occurrence matrix (GLCM) [12,13]. From the GLCM, the features of the resulting matrix were obtained for different angles (45, 90, 135, 180, and 360°), distances (1, 3), bins (5, 10, 20), window sizes (1, 2, 3), and features (1–6). From LBP, features are extracted considering different values for the radii (1 and 3) and samples (8 and 12). Overall, 7616 texture features were extracted from each patient.

Additional features considered included age, prostate volume, membranous urethral length, and Body Mass Index (BMI), which might influence continence. Therefore, they were combined with the image-derived features described above.

Supplementary Table S2 summarizes the features extracted from LA muscle for radiomics analysis. This study did not utilize feature transformation techniques because of the potential loss of meaning of the original measurements, where transformed features can be difficult to interpret and lose physical interpretation. To maintain the original meaning of the features for diagnostic and therapeutic purposes, only those features with direct interpretations in terms of shape, intensity, or texture were used.

### 2.5. Optimal Feature Selection

The complexity of using 7669 features per patient makes it difficult to determine the most significant features. We opted to develop a special approach for feature selection based on a combination of previously described methods, such as filtering, wrappers, and embedding techniques, to select the most relevant features [14]. The proposed approach is illustrated in Supplementary Figure S3. A detailed description of this method is provided in Appendix A. The sensitivity, specificity, accuracy, and area under the receiver operating characteristic (ROC) curve were computed to describe the discrimination performance of the designed model.

## 3. Results

### 3.1. Univariate Analysis and Feature Correlation

**Figure 3:**
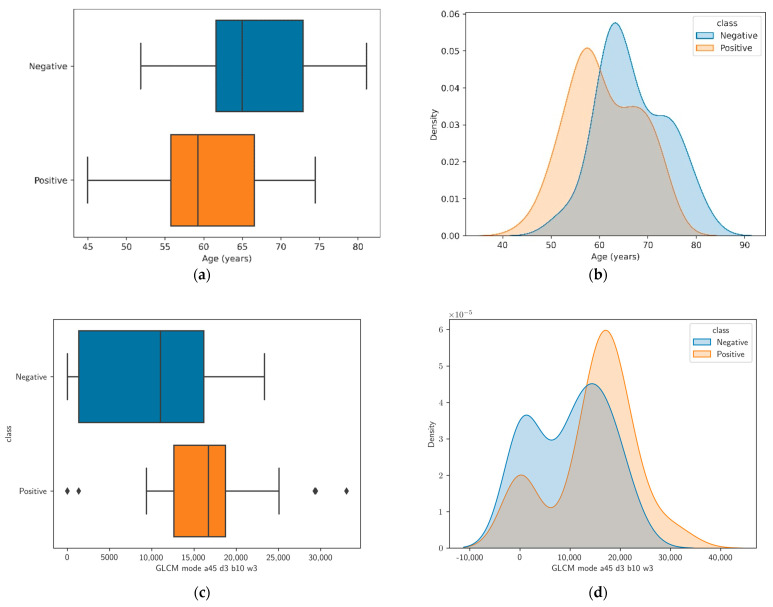
Distribution of the two features with the lowest *p*-values. (**a**) Box plot for the age feature; (**b**) Kernel density estimate for the age feature; (**c**,**d**) corresponding plots for the GLCM mode a45 d3 b10 w3 feature.

Supplementary Table S3 summarizes the univariate analysis of the features that presented the best discriminator (top ten with the lowest *p*-value) between the positive- and negative-class groups. All other features in this group were related to the texture of the LA muscle described using texture descriptors of the GLCM for different angles (a), distances (d), bins (b), window sizes (w), and features (f). Figure 3 shows the distribution of the two features with the lowest *p*-values across the two groups.

The analysis of the features in the intensity and morphology categories shows that there is insufficient evidence to conclude that there is a significant difference between the means of any of these features for the positive and negative class patients. A summary of the morphological features is presented in Supplementary Table S4, where evalue1 represents the largest eigenvalue and evalue3 represents the smallest eigenvalue. The intensity features listed in Supplementary Table S5.

**Figure 4:**
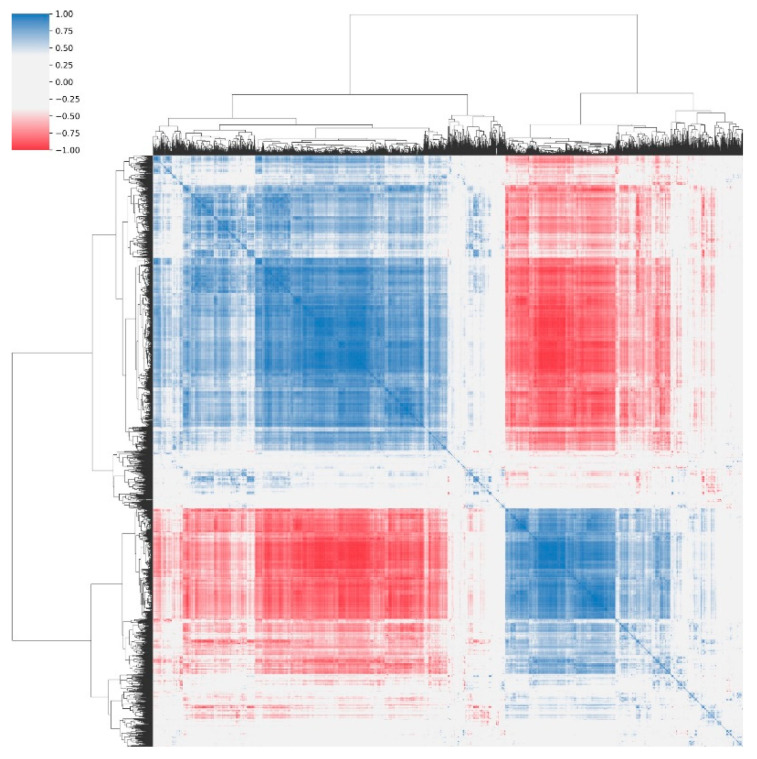
Heat map representation of feature correlations. The heat map is symmetric about the diagonal. A value of −1 means a total negative linear correlation, 0 means no correlation, and +1 means a total positive correlation.

Supplementary Table S6 summarizes the clinical features of the patients including age, prostate size, BMI, and membranous urethral length. Only age exhibited a statistically significant difference between the means of positive- and negative-class patients. Figure 4 shows a heat map representing the correlation of the features, which indicates that large groups of features are highly correlated. This indicates that these features provide redundant information, suggesting that the dimensionality of the feature space can be reduced without losing the relevant information. The OBM exploits this information.

### 3.2. Classification Performance

The number of patients in the positive and negative classes was not balanced, as 62.37% were positive. A naive classifier can classify all the patients as positive. Such a classifier produces an accuracy of 62.37% and a balanced accuracy (average between sensitivity and specificity) of 50%. A classifier built using a subset of features that provides similar metrics can be considered insignificant in terms of its discrimination capabilities.

The proposed feature selection approach was run until convergence was achieved, that is, when additional tests with new combinations of features did not improve the results. The execution was accelerated by running part of the operations on the GPU and by multi-processing. Each combination of features was evaluated by using a stratified k-fold cross-validation approach. Using this approach, the dataset is divided into five randomly chosen subsets (five folds) of roughly equal size, such that each fold preserves approximately the same class distribution. The model was trained using four subsets, and the remaining subset was used for the validation. This process was repeated five times, such that each subset was used exactly once for validation. The entire process was repeated one hundred times, and the results were averaged to improve the estimated statistics and produce robust and repeatable results.

The optimal configuration obtained using the feature selection approach includincludeded six features. Supplementary Table S7 provides a description of the six features used for the best classifier. Notably, all optimally selected features, except for age and the ratio evalue1/slambda in the right component, denote textural properties of the LA muscle. Among these features, only age was included in the top features, according to the *p*-value. Owing to redundancies, most of the top features based on the *p*-value did not survive the feature-selection process.

The resulting optimal configuration of features provided a classification accuracy of 0.84, with sensitivity, specificity, and area under the receiver operating characteristic curve of 0.90, 0.75, and 0.89, respectively. The complete performance metrics are listed in Supplementary Table S8.

Other feature selection methods in the literature were considered, including Minimum Redundancy Maximum Relevance (MRMR), Chi-square, Analysis of Variance (ANOVA), and Kruskal–Wallis [14]. Each method provides a different feature selection method. In all cases, when selecting the top ten features, the resulting classification accuracy was between 50% and 60%, which is much lower than that of the proposed feature selection approach. This corroborates the fact that the proposed feature selection approach is appropriate for this scenario.

Supplementary Table S9 shows a comparison of the performance when using different subsets of features. As expected, age alone was a good discriminating feature, providing a classification accuracy of 0.75. In contrast, BMI, which is linked to an increased risk of incontinence, provides results similar to those of the naive classifier. The group of texture features provides similar performance to the group that only uses age, with an accuracy of 0.76. Tests also indicate that features of shape or intensity properties of the LA muscle do not discriminate between continent and incontinent patients. All considered subsets are far from the performance of the optimal configuration of features that combine age, texture, and the ratio evalue1/slambda in the right component. This ratio provides relevant information for the classifier in addition to having a low *p*-value. Removing this feature decreases the accuracy to 0.81.

The same optimal configuration of features was used to train and evaluate a classifier’s ability to identify continence at 3 months. The classification performance with the same features and this different outcome decreased significantly, producing a classification accuracy of 0.66 and a balanced accuracy of 0.61. This suggests that the same optimal configuration of features can discriminate between positive and negative class patients at a 12-month time point, but not at a 3-month time point.

## 4. Discussion

Over the years, there has been an improvement in our understanding of the surgical anatomy of the prostate gland, accompanied by the adoption of several surgical modifications for RARP to improve functional outcomes [2,5,15]. However, urinary incontinence is a major drawback of this surgery. Several patient-related factors have been linked to increased odds of incontinence after RARP, such as increased age, BMI, prostate volume, and levator ani morphology characteristics, namely thickness [2,6].

In this study, we identified MRI-derived radiomic features of the LA muscle component of the urogenital hiatus in a cohort of men who underwent robot-assisted prostatectomy. Our approach, which accounts for the volumetric characteristics of the LA muscle in 3D space rather than relying solely on single-slice analysis, introduces a novel dimension to evaluate alterations within this critical anatomical region. The consideration of multiple layers of muscle tissue aids in capturing tissue heterogeneity and structural variations, making the selected radiomic features more robust and informative. We proposed a novel model to select features extracted from the LA muscle on T2-weighted MRI images that can classify post-prostatectomy patients into continent and incontinent patients at 12 months. With a 97-patient training set and features from T2-weighted MRI sequences alone, our optimum selected features demonstrated a discriminatory sensitivity, specificity, accuracy, and area under the ROC curve of 0.90, 0.75, 0.84, and 0.88, respectively. The proposed model is highly effective at handling many features in a complex search space, selecting the most relevant and potent features, while ensuring stable and repeatable results with a low number of samples. By combining statistical significance and randomness to find good groups of features and evaluating each group of features using a classifier and a repeated stratified K-fold cross validation, the proposed approach can select the most relevant features, resulting in improved accuracy compared to other methods in the literature [14].

Our study thoroughly evaluated different classification algorithms, including Decision Trees, Naive Bayes, Support Vector Machines, Nearest Neighbor, XGBoost and Neural Network. All these methods were considered in the Bayesian optimization approach [16]. Among these methods, the most promising results were achieved using the XGBoost algorithm, a robust machine-learning technique within the gradient-boosting framework. Gradient boosting is a potent and extensively utilized machine learning approach acclaimed for its exceptional predictive prowess. Operating within the framework of ensemble learning, Gradient Boosting constructs a series of models sequentially, each one dedicated to correcting the mistakes of its predecessors. The algorithm methodically hones its predictions by progressively addressing misclassified instances, culminating in a resilient and precise model. This initial methodology amplifies predictive precision and empowers the algorithm to navigate intricate data relationships adeptly.

The findings of our study are in line with those of previous studies that showed a correlation between age and the postoperative continence rate [1,2]. However, a recent study showed that LA muscle thickness is correlated with urinary continence after RARP [6]. In contrast, we found that LA muscle textural features and handcrafted features such as the ratio evalue1/slambda in the right component (which roughly indicates elongatedness of the right component) were able to identify continent patients at 12 months, and that none of the morphological and intensity-derived features, such as LA muscle thickness and kurtosis, were correlated with continence. In the present study, several complex LA textural features were valuable for differentiating between the continent and incontinent groups. The GLCM features describe the second-order statistical information of the gray levels between neighboring pixels in an image. Several studies showed that these textural properties have been proposed to reflect concealed pathomorphological texture patterns [17,18].

The notable correlation between particular LA muscle textural features and hand-crafted features and continence outcomes reveals the potential of radiomics to provide unique insights into the complexity of post-RARP urinary continence. Interestingly, our study did not demonstrate a significant correlation between continence outcomes and traditional morphological and intensity-derived features such as LA muscle thickness and kurtosis. This underscores the need to explore beyond conventional measurements and consider more intricate textural properties that might indicate underlying pathophysiological changes.

The non-survival of certain features suggests a high correlation with other surviving features. This phenomenon is particularly relevant in complex datasets with intricate interrelationships among the variables. As depicted in the figure with feature correlation, strong correlations among features become evident, underscoring the potential influence of multicollinearity within the dataset. The observed correlations among the features indicate that they might collectively capture similar underlying information or share common aspects. In the feature selection process, this correlation can affect the significance attributed to individual attributes. When highly correlated features are present, they might collectively contribute to the discrimination task, leading to the selection of one feature, whereas others are omitted. This outcome aligns with the principle of favoring simplicity and avoiding redundancy in the model, as overly correlated features might not provide additional meaningful insights. Recently, a distinct radiomic phenotype/signature was identified, capturing the association between muscle heterogeneity detected by ultrasound radiomic features and the incidence of hearing impairment, stroke, myocardial infarction, dementia, frailty, and falls among aging patients [19]. Moreover, the authors theorized that the radiomic phenotype of muscle dysfunction could be due to mitochondrial dysfunction, which affects many cellular processes involved in energy metabolism [19,20]. The results of our study might be interpreted within the framework of this theory, as age might induce textural or heterogeneity changes in the LA muscle that reflect the underlying functional aspects of tissue changes.

This model may help physicians to preoperatively identify patients who may become incontinent after RARP. Consequently, this could potentially improve patient counseling, facilitate identification of ideal candidates for surgery, and help patients to consider alternative treatment options when radiomic features associated with urine incontinence post-RARP are present. Our study has several limitations, including a relatively small sample size from a single institution, a retrospective study design, and an imbalance between the outcome classes, given that a substantial number of patients recover within 12 months of surgery. Moreover, the analysis did not account for pre-existing lower urinary tract symptoms and nerve-sparing status, as most of our patients underwent nerve sparing surgery. Finally, a reproducibility analysis of LA muscle contouring was not performed in this study, although LA muscle segmentation was checked by a board-certified radiologist (DAT). Although this approach has not been externally validated, the results presented herein are promising.

## 5. Conclusions

In conclusion, our study pioneered a novel radiomic approach utilizing LA muscle radiomic features from T2-weighted MRI images to predict continent and incontinent patients at 12 months post RARP By focusing on the volumetric representation of the LA muscle, our study transcends conventional single-slice assessment, potentially enhancing the precision of continence prediction. These results mark an important first step towards advancing personalized patient care, as our model could aid clinicians in identifying patients at increased risk of incontinence, facilitating tailored treatment plans, and improving informed patient counseling.

## Data Availability

Data are available upon request from the reviewer.

## Supplementary Materials

The following supporting information can be downloaded at: https://www.mdpi.com/article/10.3390/diagnostics13182913/s1, Figure S1: Consort Diagram: A Visual Representation of Study Participant Flow and Analysis; Figure S2: 3D representation of the equivalent ellipsoids for the left and right components of the levator ani muscle; Figure S3: Workflow of the proposed approach, integrating Multiparametric Magnetic Resonance Imaging (mpMRI), delineation of the Region of Interest (ROI) and Optimal Biomarker Method (OBM); Table S1: Summary of patient cohort characteristics; Table S2: Summary of the features; Table S3: Univariate analysis of features sorted by *p*-value (top 10). The table shows the mean value of each feature for the positive and negative patients, the variability of the means and the *p*-value; Table S4: Univariate analysis of features in the category morphologic sorted by *p*-value (top 10); Table S5: Univariate analysis of features in the category intensity sorted by *p*-value (top 10); Table S6: Univariate analysis of features in the clinical category sorted by *p*-value; Table S7: Optimal subset of features; Table S8: Performance of the optimal subset of features; Table S9: Performance of different subsets of features.

## Appendix A. Feature Extraction, Selection, and Classification Algorithms

The proposed methodology commences by extracting a comprehensive set of quantitative attributes from a designated region of interest within the 3D MRI volume. These attributes are categorized into four distinct groups: morphology, intensity, texture, and clinical variables. The latter comprises data such as age, prostate size, body mass index (BMI), and urethral length.

Morphological features encapsulate information about the shape, size, and spatial arrangement of the region of interest within the volumetric data. Global characteristics encompass both volume and surface area metrics. Subsequently, the attributes pertaining to each of the two components of the levator ani (LA) muscle, specifically the left and right components, are computed. These components correspond to the two largest connected regions within the binary 3D volumetric image. For each of these components, key metrics including volume, surface area, and eigenvalues derived through Principal Component Analysis (PCA) of the 3D object volume are determined. The eigenvalues are proportional to the squares of the lengths of the equivalent ellipsoid axes for each component, rendering them valuable approximations of the volume’s shape characteristics. The mathematical justification for why the eigenvalues are proportional to the squares of the lengths of the equivalent ellipsoid axes comes from the fundamental principles of linear algebra and the geometric interpretation of eigenvalues and eigenvectors. When PCA is applied to a 3D object, the eigenvectors represent the principal axes of the object’s distribution, and the eigenvalues represent the variances along these axes. In the context of equivalent ellipsoids, the eigenvectors correspond to the axes of the ellipsoid, and the eigenvalues represent the variances or the “spread” of the data along these axes. Mathematically, the proportionality between eigenvalues and the squares of the lengths of the equivalent ellipsoid axes can be expressed as follows:

- Let λ1, λ2, and λ3 be the eigenvalues obtained from PCA.
- Let a, b, and c be the lengths of the equivalent ellipsoid’s major, intermediate, and minor axes, respectively.
- The relationship is: λ1 ∝ a^2^ λ2 ∝ b^2^ λ3 ∝ c^2^.

This proportionality arises because the eigenvalues are directly related to the variances along the corresponding axes, and the variances are proportional to the squares of the lengths of those axes.

Additional morphological features are also derived, encompassing ratios involving the eigenvalues, as well as comparisons between the left and right components. In total, thirty-five morphological attributes are computed for each subject.

Intensity features encompass a set of statistical metrics characterizing the intensity or luminance values within the LA muscle voxels. These metrics encompass properties such as mean, median, standard deviation, mode, maximum, minimum, quartiles, moments, skewness, kurtosis, and peak height. In total, fourteen intensity features were computed for each subject.

Texture features were developed to quantify the perceived texture, or heterogeneity, within the LA muscle. Two distinct types of texture descriptors were employed: the Local Binary Pattern (LBP) and the Gray-Level Co-occurrence Matrix (GLCM).

The Local Binary Pattern (LBP) is a texture analysis method used in image processing and computer vision to describe the texture of an image or a region within an image. LBP is particularly useful for characterizing the local patterns and textures in an image, making it valuable for various applications such as face recognition, texture classification, and object detection. The LBP method operates on grayscale images or volumes. It defines a local pattern at each pixel by comparing the pixel’s intensity to the intensities of its neighboring pixels. The core idea is to convert a neighborhood of pixels into a binary pattern, where each neighbor is compared to the central pixel. The result is a binary number that represents the local texture pattern. For each pixel in the neighborhood, the intensity value is compared to the intensity of the central pixel. If the neighbor’s intensity is greater or equal to the central pixel’s intensity, it is assigned a binary ‘1’, otherwise a ‘0’. This process is repeated for all pixels in the neighborhood. The binary results are concatenated or arranged in a specific order (e.g., clockwise or counterclockwise) to create a binary pattern. This binary pattern represents the local texture around the central pixel. Once the binary patterns are calculated for all pixels in the image, a histogram of these patterns is created. The histogram characterizes the distribution of local texture patterns in the image.

The Gray-Level Co-occurrence Matrix (GLCM) is a texture analysis method used in image processing and computer vision to quantify the spatial relationships between pixel values in an image. GLCM captures the joint occurrence of pairs of pixel values at a certain relative position or distance within an image, providing valuable information about the texture, structure, and patterns present in the image. The GLCM method is based on the statistical properties of pixel pairs in an image. It involves calculating a matrix that represents the frequency of occurrence of each pair of pixel values at a specific relative position or distance within the image. For each pixel in the image, the relative position or distance and direction within the image is defined. This defines the neighborhood of interest for capturing texture information. For each pixel in the image, the neighbors are examined within the defined distance and direction. Pairs of pixel values are created by considering the intensity value of the central pixel and the intensity value of its neighbor. These pairs are then used to populate an *n* × *n* co-occurrence matrix (where *n* is the number of distinct gray levels in the image). Each element (i, j) in the matrix represents the number of times a pair of pixel values with intensities i and j occur at the specified relative position.

Additionally, age, prostate volume, membranous urethral length, and BMI clinical variables that could impact continence, were combined with the previously described image-derived features.

Feature selection involves choosing a group of relevant features to include in building a classification model. This improves the model’s accuracy and makes it easier to understand by decreasing the number of features and removing unnecessary, redundant, or irrelevant ones. A basic method would be to test all possible combinations of features, but with 7669 features, this would take an impractically long time (102,284 billion years). Thus, a more efficient feature selection method is necessary.

There are several methods for feature selection, which can be broadly categorized into three main types: filter methods, which use statistical measures to evaluate the relevance of each feature; wrapper methods, which use a learning algorithm to evaluate the performance of different feature subsets; and embedded methods, which learn the optimal feature subset as part of the model training process [21]. Existing methods differ in training times as well as stability, with no single method consistently outperforming the others [22].

The proposed approach in this work for feature selection is a combination of these methods to select the most relevant features. The proposed approach is designed to provide stable and repeatable results with a small number of samples. First, groups of features are selected that satisfy two conditions: (i) they are as minimally correlated among all features as possible, and (ii) they hold the best power to discriminate between the two classes. These conditions are evaluated on a randomly selected training subset with 60% of the samples. This stochastic approach combines statistical significance and randomness to find good groups of features. This method is highly effective in tackling this issue, as it handles a considerable number of features in a complex search space.

The procedure starts by randomly dividing the dataset into three groups: 60% for training, 20% for validation, and 20% for testing. Then, using the training subset, the class distance (d) of each image-based feature (f) between positive and negative subjects is calculated using Equation (A1), where $\mu_{pos}$ and $\mu_{neg}$ are the mean values of the feature for positive and negative subjects and $\sigma_{pos}$ and $\sigma_{neg}$ are the variances of the features for positive and negative subjects, respectively.

(A1)
$$d\left(f\right)=\frac{\mu_{pos}\left(f\right)-\mu_{neg}\left(f\right)}{\sqrt{\sigma_{pos}\left(f\right)\sigma_{neg}\left(f\right)}}$$

Next, the correlation from each feature to all the others is computed. Only features with a correlation ratio lower than a threshold ρ are considered to be uncorrelated. A parameter θ is also defined to describe the fraction of the total number of uncorrelated features with f. Then, a combined distance metric (D) is defined for each feature as a function of d, ρ,  and θ to describe the distance between positive and negative subjects for feature f. The combined distance metric is defined using Equation (A2).

(A2)
$$D\left(f,d,\rho \right)=\;\left\{\begin{array}{cc} \sqrt{d^{2}\left(f\right)+{\left(1-\rho \right)}^{2}+\theta^{2}\left(f,\rho \right)} & if\;\theta \geq 0.5\;\mathrm{and}\;p\;\mathrm{value}\;\leq \;0.05\\ 0 & if\;\theta <0.5\;\mathrm{and}\;p\;\mathrm{value}\;>\;0.05 \end{array}\right.$$

The combined distance metric is only calculated for subjects in the training subset. The distance for a feature f is zero when either the *t*-test *p* value is greater than 0.05 or the fraction of features having low correlation with the given feature is lower than 0.5. Otherwise, the distance is calculated as the square root of the three terms indicated in Equation (A2).

Feature selection is initially applied based on the combined distance metric. Features are sorted in descending order according to the combined distance metric. Only the top Nf is selected, where Nf is a configurable parameter of the procedure. In case two features have the same value for the combined distance metric, the feature having the larger class distance, smaller ρ, and larger  θ is selected.

Each group of features is evaluated using a classifier following a repeated (one hundred times) stratified K-Fold cross validator. This procedure is repeated, producing the best candidate group of features. Then, all possible combinations of features in this group taking 1, 2, … features at a time are evaluated using the same approach. The final step is the computation of a classification model using Bayesian optimization for the best combination, which results in the best classification model and optimal hyperparameters for the model and the considered problem. Bayesian optimization is the selected approach for the final computation of the model, as it provides a major advantage over other hyperparameter optimization techniques [23]. The sensitivity, specificity, accuracy, and area under the receiver operating characteristic (ROC) curve are computed to describe the discrimination performance of the designed model. The considered Bayesian optimization approach determines the best model and hyperparameters considering the most common classification algorithms used in the literature: Decision Tree, Naive Bayes, Support Vector Machine, Nearest Neighbor, XGBoost, and Neural Network. Among all of the classification algorithms considered, XGBoost consistently demonstrated superior performance in the experiments.

XGBoost, which stands for “eXtreme Gradient Boosting”, is a powerful and widely used machine learning algorithm for both classification and regression tasks. It belongs to the ensemble learning family, specifically gradient boosting, and is known for its exceptional performance in various machine learning competitions and real-world applications. XGBoost is based on the gradient boosting framework. Gradient boosting is an ensemble learning technique that combines the predictions of multiple weak learners (typically decision trees) to create a strong predictive model. It minimizes a loss function by iteratively adding weak learners, and each new learner is trained to correct the errors made by the existing ensemble. XGBoost formulates the learning problem as an optimization task. It defines an objective function (also known as the loss function) that quantifies the difference between the predicted and actual target values. The objective function consists of two parts: a data loss term that measures the model’s accuracy, and a regularization term that discourages overly complex models to prevent overfitting. To optimize the objective function, XGBoost uses gradient descent techniques. XGBoost’s success lies in its ability to combine gradient boosting with various algorithmic optimizations, regularization techniques, and feature engineering to produce accurate and efficient machine learning models.
