# Supplementary material for: MRI-Based Radiomics Analysis of Levator Ani Muscle for Predicting Urine Incontinence after Robot-Assisted Radical Prostatectomy
Source: Diagnostics (Basel). 2023 Sep 11;13(18):2913. doi: 10.3390/diagnostics13182913 (PMC10528635; doi:10.3390/diagnostics13182913)
Supplement: Supplementary file 1 [file diagnostics-13-02913-s001.zip › diagnostics-2525178-supplementary.pdf]

diagnostics

Supplementary Material: MRI-Based Radiomics Analysis of Levator Ani Muscle for Predicting Urine Incontinence After Robot-Assisted Radical Prostatectomy

Mohammed Shahait, Ruben Usamentiaga, Yubing Tong, Alex Sandberg, David I. Lee, Jayaram K. Udupa, and Drew A. Torigian

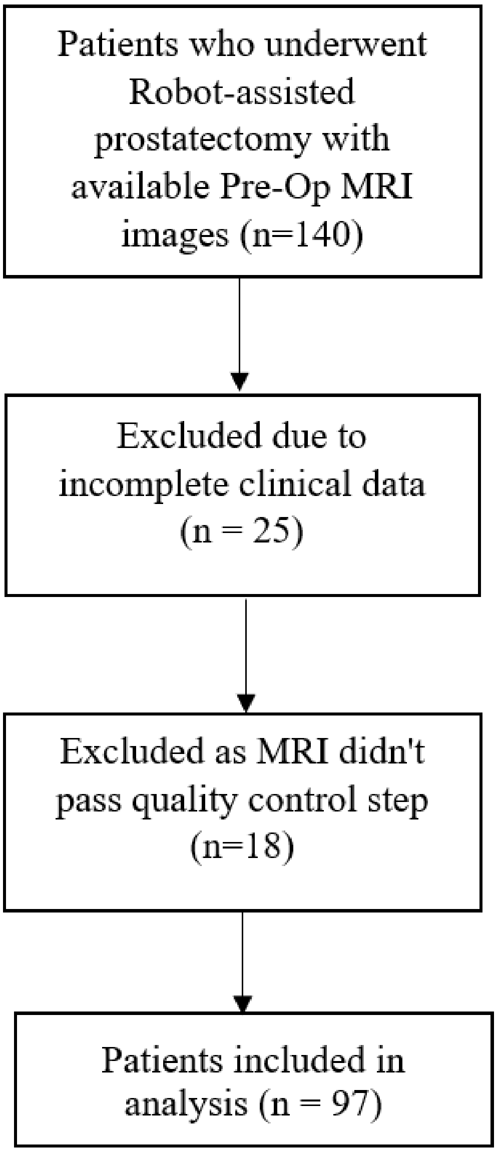

Figure S1. Consort Diagram: A Visual Representation of Study Participant Flow and Analysis.

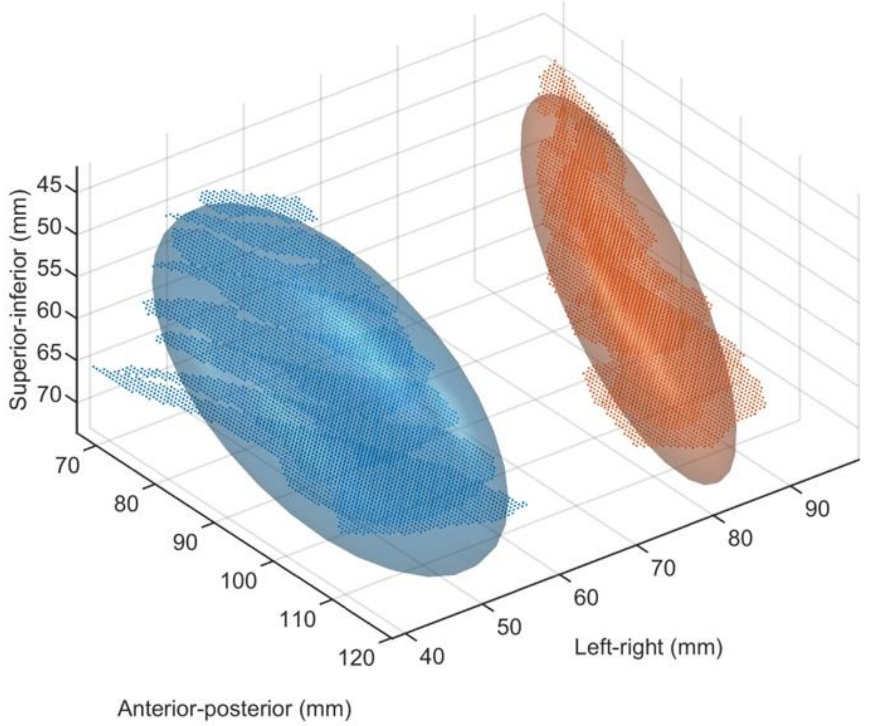

Figure S2. 3D representation of the equivalent ellipsoids for the left and right components of the levator ani muscle.

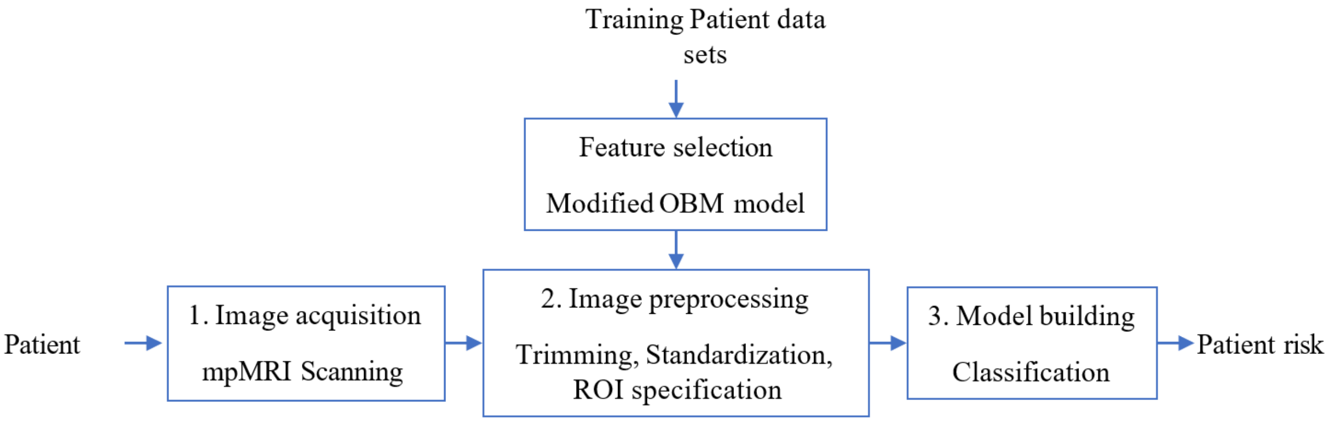

Figure S3. Workflow of the proposed approach, integrating Multiparametric Magnetic Resonance Imaging (mpMRI), delineation of the Region of Interest (ROI) and Optimal Biomarker Method (OBM).

Table S1. Summary of patient cohort characteristics.

Variable		Overall N=97
Age (years)	Median	63.5
	IQR	57.7-69.9
BMI (kg/m2)	Median	27.8
	IQR	25.0-32.0
American Urological Association Symptom Index	Median	7
	IQR	3.75-13
Preoperative PSA (ng/mL)	Median	5.7
	IQR	4.4-8.2
Prostate Size (ml)	Median	40.5
	IQR	28.6-56.7
Pathological Gleason Score	6	37
	7	53
	8	4
	≥9	3
Surgical Margin	Positive	22
	Negative	75
Pathological T Stage	≤ T2c	66
	T3a	26
	T3b	5
Membranous urethral length	Mean	13.65
	+SD	2.13
Nerve Sparing		
	Full Nerve Sparing *	95
	Partial Nerve sparing	1
	No Nerve sparing	1

*(at least on one side) (BMI: Body Mass Index; IQR: Interquartile Range; PSA: Prostate-Specific Antigen).

Table S2. Summary of the features.

Category	Number of features	Description
Morphologic	35	Volume, surface area, eigenvalues, ratios, . . .
Intensity	14	Max, min, std, mean, median, mode, moments, . . .
Texture	7616	Features from local binary patterns and co-occurrence matrix
Clinical	4	Age, prostate volume, urethral length, and body mass index
Total	7669	

Table S3. Univariate analysis of features sorted by p-value (top 10). The table shows the mean value of each feature for the positive and negative patients, the variability of the means and the p-value.

Feature	Mean (pos.)	Mean (neg.)	Std (pos.)	Std (neg.)	p-value
Age	60.94	66.80	7.22	6.96	0.0004
GLCM mode a45 d3 b10 w3 f5	14716.40	9941.45	8312.35	7518.14	0.0087
GLCM mode a360 d3 b20 w3 f3	1375.06	1484.30	166.19	212.29	0.0093
GLCM skewness a180 d3 b10 w1 f3	288.65	271.52	27.65	32.47	0.0107

GLCM minimum a135 d1 b10 w3 f1	1613.42	1661.67	83.30	88.47	0.0126
GLCM mode a360 d1 b10 w2 f5	17409.83	11933.45	9379.52	10205.31	0.0127
GLCM maximum a360 d3 b20 w1 f2	1863.64	1858.58	9.48	8.14	0.0129
GLCM mode a45 d3 b5 w3 f5	23792.34	16974.15	11199.85	13953.88	0.0145
GLCM kurtosis a180 d3 b10 w1 f3	168043.38	156074.23	19802.32	24487.44	0.0149
GLCM minimum a360 d3 b20 w1 f1	1032.83	1048.18	30.40	23.75	0.0156

(GLCM: Gray-Level Co-occurrence Matrix).

Table S4. Univariate analysis of features in the category morphologic sorted by p-value (top 10).

Feature	Mean (pos.)	Mean (neg.)	Std (pos.)	Std (neg.)	p-value
Ratio surface left/right	1.06	1.02	0.09	0.10	0.0506
Ratio volume left/right	1.07	1.01	0.20	0.15	0.1068
Ratio evalue3/slambda left	0.42	0.40	0.14	0.12	0.3718
Ratio slambda left/right	1.01	1.00	0.06	0.07	0.4080
Evalue1 right	113.51	119.47	33.60	41.17	0.4653
Evalue3 left	5.28	5.02	1.77	1.49	0.4850
Slambda right	12.43	12.70	1.67	1.86	0.4876
Evalue3 right	5.01	5.23	1.56	1.39	0.5068
Ratio surface/volume left	0.68	0.70	0.09	0.11	0.5075
Ratio evalue2 left/right	1.11	1.08	0.20	0.26	0.5299

(evalue1 represents the largest eigenvalue and evalue3 the smallest eigenvalue).

Table S5. Univariate analysis of features in the category intensity sorted by p-value (top 10).

Feature	Mean (pos.)	Mean (neg.)	Std (pos.)	Std (neg.)	p-value
Third moment	83381.72	54425.30	82317.34	45273.54	0.0676
Skewness	43.58	37.32	17.22	12.92	0.0761
Fourth moment	1.20e+08	6.78e+07	1.61e+08	7.73e+07	0.0862
Second moment	139.68	117.51	72.20	59.19	0.1425
Kurtosis	4596.48	3806.07	3149.53	1820.83	0.1938
Maximum	2239.60	2067.21	729.49	618.27	0.2626
STD	228.13	209.18	76.28	75.60	0.2644
Mode	277.19	299.70	107.46	154.35	0.4278
Peak	1.17	1.11	0.41	0.49	0.5672
Low quartile	251.55	262.58	77.69	113.27	0.5937

(STD: Standard Deviation)

Table S6. Univariate analysis of features in the clinical category sorted by p-value.

Feature	Mean (pos.)	Mean (neg.)	Std (pos.)	Std (neg.)	p-value
Age	60.94	66.80	7.22	6.96	0.0004
Membranous urethral length	13.86	13.44	1.84	1.57	0.2723
Prostate size	47.20	41.63	28.79	26.16	0.3692
BMI	30.03	30.26	6.58	5.81	0.8717

Table S7. Optimal subset of features.

Feature
1: Age
2: Ratio evalue1/slambda right
3: GLCM mode a180 d1 b10 w3 f5
4: GLCM maximum a45 d3 b20 w1 f3
5: GLCM maximum a45 d3 b20 w1 f1
6: GLCM minimum a360 d1 b10 w1 f3

Table S8. Performance of the optimal subset of features.

Metric	Value
Accuracy	0.843
Balanced accuracy	0.832
Recall	0.906
Precision	0.865
F1	0.864
ROC AUC	0.885
Sensitivity	0.906
Specificity	0.758

(F1: F1 score; ROC: Receiver Operating Characteristic; AUC: Area Under the Curve).

Table S9. Performance of different subsets of features.

Subset of features	Accuracy	Balanced accuracy
Optimal	0.843	0.832
All in the clinical category	0.767	0.742
All in the morphologic and intensity category	0.615	0.531
All in the texture category	0.762	0.740
Only the age feature	0.756	0.742
Only the BMI feature	0.618	0.496
Optimal without ratio evalue1/slambda right	0.816	0.800
